# Prognostic and immunological significance of an M1 macrophage-related gene signature in osteosarcoma

**DOI:** 10.3389/fimmu.2023.1202725

**Published:** 2023-07-03

**Authors:** Xiaoyu Mao, Fanglong Song, Ju Jin, Bin Zou, Peijun Dai, Mingjuan Sun, Weicheng Xu, Lianghua Wang, Yifan Kang

**Affiliations:** ^1^ Department of Orthopedics, Third Affiliated Hospital of Naval Medical University, Shanghai, China; ^2^ Department of Orthopedics, The Second Affiliated Hospital of Soochow University, Suzhou, Jiangsu, China; ^3^ Department of Biochemistry and Molecular Biology, College of Basic Medical, Naval Medical University, Shanghai, China; ^4^ Department of Traditional Chinese Medicine, Dujiangyan Air Force Special Service Sanatorium, Chengdu, Sichuan, China

**Keywords:** osteosarcoma, macrophage-related genes, transcriptomics analysis, prognostic signature, tumor microenvironment, immunotherapy

## Abstract

As the most abundant infiltrating immune cells in the tumor microenvironment (TME), tumor-associated macrophages (TAMs) are pivotal in tumor development and treatment. The present investigation endeavors to explore the potential of M1 macrophage-related genes (MRGs) as biomarkers for assessing risk in individuals with osteosarcoma. RNA-sequence data and clinical data were derived from TCGA and GEO databases. The CIBERSORT method was utilized to discern subtypes of tumor-infiltrating immune cells. Identification of MRGs was achieved through Pearson correlation analysis. A prognostic risk model for MRGs was developed using Cox and LASSO regression analyses. A tripartite gene signature comprising CD37, GABRD, and ARHGAP25 was an independent prognostic indicator and was employed to develop a risk score model. The internal and external validation cohort confirmed the results. The area under the ROC curve (AUC) was determined for survival periods of 1 year, three years, and five years, yielding values of 0.746, 0.839, and 0.850, respectively. The C-index of the risk score was found to be superior to clinicopathological factors. GO/KEGG enrichment showed that the differences between high- and low-risk groups were predominantly associated with immune response pathways. Immune-related analysis related to proportions of immune cells, immune function, and expression levels of immune checkpoint genes all showed differences between the high- and low-risk groups. The qRT-PCR and Western blotting results indicate that CD37 expression was markedly higher in MG63 and U2OS cell lines when compared to normal osteoblast hFOB1.19. In U2OS cell line, GABRD expression levels were significantly upregulated. ARHGAP25 expression levels were elevated in both 143B and U2OS cell lines. In summary, utilizing a macrophage genes signature demonstrates efficacy in predicting both the prognosis and therapy response of OS. Additionally, immune analysis confirms a correlation between the risk score and the tumor microenvironment. Our findings, therefore, provide a cogent account for the disparate prognoses observed among patients and furnish a justification for further inquiry into biomarkers and anti-tumor treatment strategies.

## Introduction

Osteosarcoma (OS) is a widely occurring primary bone tumor that stems from primitive mesenchymal cells (1). This type of tumor primarily affects the long bones, where the sarcoma cells create immature bone or osteoid tissues (2). Nearly two-thirds of all primary bone malignancies are accounted for by OS, which is the most prevalent bone cancer among children and adolescents (3). Essential features include severe pain and swelling of the impacted bones; in some instances, osteosarcoma may result in pathological fractures. OS can potentially spread to various body parts, most notably the lungs (4). The survival rates for two years, 5 years, and ten years are 67%, 49%, and 42%, respectively (5). The overall survival for patients with metastases is poor, ranging from 15% to 30% (6). Due to the complicated and unsteady nature of the genome, the effects on treatment outcomes are substantial (7). Therefore, it is necessary to identify novel prognostic gene markers to predict the prognosis of OS and guide the treatment regimens.

The treatment for osteosarcoma has evolved from amputation to preserving limbs. Lately, immunotherapeutic approaches, including adoptive cell treatments, vaccinations, and immune checkpoint inhibitors, have become potential therapeutic strategies (8). Preclinical studies have demonstrated encouraging results for OS with immunotherapy (9, 10). However, the objective response for OS patients receiving anti–programmed cell death 1 antibody remains unsatisfactory (11, 12). The low rate of response could be attributed primarily to the heterogeneity of the tumor immune microenvironment (TME). This is due to the distinct subsets of immune cells that perform contrasting roles in either promoting or inhibiting tumorigenesis (13).

Tumor-associated macrophages (TAMs), which are the predominant infiltrating immune cells within the tumor microenvironment, exhibit the capacity to undergo phenotypic polarization. This process is driven by specific cues from the surrounding microenvironment, facilitating the initiation of tailored functional responses (14). Macrophage phagocytosis could result in the eradication of tumors, the initiation of inflammasome activation, and the presentation of antigens. These processes may stimulate the development of adaptive immunity (14). During the initial stages of tumor development, TAMs predominantly exhibit an M1 pro-inflammatory phenotype and facilitate immune reactions that restrain tumor growth. With tumor progression, TAMs undergo a gradual transition towards an M2 functional phenotype that promotes their involvement in tumor angiogenesis and immunosuppression (15, 16). M1 macrophages demonstrate anti-tumor activity via the synthesis of pro-inflammatory molecules including tumor necrosis factor alpha (TNF-α), interleukin-1 beta (IL-1β), and inducible nitric oxide synthase (iNOS), as well as the secretion of chemokines such as C-X-C motif chemokine ligand 10 (CXCL10), C-X-C motif chemokine ligand 11 (CXCL11), and C-C motif chemokine ligand 2 (CCL2). Furthermore, M1 macrophages exhibit the presence of antigen-presenting molecules (MHCII), co-stimulatory molecules, and antigen-processing peptidases, all of which play a role in enhancing their anti-tumor capabilities (17).. Macrophage-related genes have been reported to correlate with the prognosis, and immunotherapy response in kinds of tumors, which suggests that MRGs have acceptable prognostic values for disease outcomes (18–22). However, the role of M1 macrophages related genes in the prognosis of OS and treatment response has not been well studied.

Here, this study is to explore the potential of M1 macrophage-related genes as biomarkers for assessing risk in individuals with osteosarcoma. By comparing gene expression patterns between high- and low-risk groups, we analyzed differentially expressed genes (DEGs) and investigated the underlying molecular mechanisms, regulatory pathways, and immune cell infiltration. The main objective of this study was to elucidate the immunogenomic profile of osteosarcoma and identify survival-associated genes that could serve as valuable clinical biomarkers and guide treatment plans.

## Materials and methods

### Data collection and processing

The mRNA expression data and clinical details for osteosarcoma patients (TARGET-OS dataset) were obtained from the Cancer Genome Atlas Program (TCGA) database (https://portal.gdc.cancer.gov/). The RNA-seq raw read count from the TCGA database was converted to transcripts per kilobase million (TPM) and further log-2 transformed. The GSE21257 dataset from the Gene Expression Omnibus (GEO) database (https://www.ncbi.nlm.nih.gov/geo/) supplied as an external validation set mRNA expression data and clinical information for osteosarcoma specimens. The CIBERSORT algorithm was employed to quantify the presence of 22 infiltrating immune cell types within the TME using the TARGET-OS dataset (23). Pearson correlation analysis identified 281 genes exhibiting a correlation with M1 macrophage expression (|R2| > 0.4, p < 0.001). Samples with a survival duration of less than 30 days were excluded from the analysis.

### Development of prognostic genes signature

A total of 84 samples, consisting of survival and expression data from the TARGET-OS dataset, were divided into a training set (n=59) and an internal validation set (n=25) at a 7:3 ratio. The GSE21257 dataset provided 53 samples for an external validation cohort. In the training set, univariate Cox regression analysis identified M1 macrophage genes with prognostic relevance. The least absolute shrinkage and selection operator (LASSO) algorithm, with 1000 iterations, was then employed to select the optimal subset of prognostic genes, leading to the development of an M1 macrophage gene signature (MRS). The multivariate Cox regression model determined the final genes after LASSO algorithm application. Risk scores were calculated using the linear combination of each chosen gene, following the formula: Risk score = ∑(coef (β) * EXP(β)), where β denotes the regression coefficient. Patients were categorized into high- and low-risk groups based on the median risk score as the threshold, and the clinical differences between these groups were investigated using Kaplan-Meier survival analysis. The predictive accuracy of the model was evaluated via the ROC curve and C-index. Moreover, stratified analysis was performed to assess the additional prognostic value of the MRS.

### Validation of the MRS

The patients of the internal and external validation set were subjected to the identical grouping methodology utilized in the training set, after which their survival was assessed through the application of Kaplan-Meier survival analysis and risk plot.

### Construction of nomogram

A nomogram was constructed to forecast the 1-, 3-, and 5-year survival rates of OS patients using the risk score in conjunction with the clinicopathological factors of age, gender, race, and metastasis. The accuracy of the nomogram’s predictions was then tested using a calibration curve to compare actual overall survival with predicted survival rates.

### Functional enrichment analysis in the TARGET-OS cohort

The cohort was partitioned into high- and low-risk groups using the predetermined risk score threshold. Subsequently, gene expression fold changes were analyzed using the R package “limma”. Pathway analysis was performed using the R package “clusterProfiler” and focused on identifying significantly enriched pathways within the reference gene set for the high- and low-risk groups. The reference gene set was defined as the hallmark gene sets described by Subramanian et al. (24).

### Immune-related analysis of MRS

Single-sample gene set enrichment analysis (ssGSEA) algorithm using R packages, specifically limma, GSVA, and GSEABase, was used to evaluate the disparity in immune function between high- and low-risk groups classified according to MRS (25). TME and immune cell infiltration were evaluated using the ESTIMATE and CIBERSORT algorithms to determine the proportions of its components (23, 26). Subsequently, the association between the expression levels of immune checkpoint genes and the two groups was investigated.

### Significance of the MRS in drug sensitivity

The Genomics of Drug Sensitivity in Cancer (GDSC) public repository offers valuable insights into cancer cell drug sensitivity and associated molecular markers for drug responses (27). By employing the oncoPredict package, gene expression profiles from GDSC2 and corresponding drug response data were obtained to build a ridge regression model tailored for osteosarcoma transcriptomic information. Following this, sensitivity scores were calculated to estimate the half-maximal inhibitory concentration (IC50) for various drugs in the context of OS patients.

### Cell culture

Human OS cell lines (143B, MG63, and U2OS) and human normal osteoblast cell line (hFOB1.19) were purchased from Wuhan Servicebio Technology Co., Ltd. (Wuhan, China). Each cell line was cultured in its specific medium (Wuhan Servicebio Technology Co. Ltd., Wuhan, China). Human OS cell lines and hFOB1.19 cells were cultured, respectively, at 37°C in humidified incubator with 5% CO_2_ and at 34°C in an incubator with 5% CO_2_.

### RNA extraction and qRT-PCR

Total RNA was extracted from OS cell lines and the normal osteoblast cell line, hFOB1.19, employing TRIzol reagent (Invitrogen, USA). First-strand cDNAs were synthesized using the PrimeScript™ RT reagent kit (Takara, Japan) as per the manufacturer’s guidelines. MRG mRNA levels were assessed through qRT-PCR, utilizing SYBR Premix Ex Taq (TaKaRa, Japan) and specific primers (**Table 1**). The relative expression levels were determined and adjusted to the reference control GAPDH using the 2^–ΔΔCt^ method.

**Table 1:** Primer sequence for qRT-PCR.

Primer name	Sequence
CD37 NM_001040031	F: 5′-GCTGGGACTATGTGCAGTTCC-3′ R: 5′-ACCCGTTACCTCTCAGGATGA-3′
ARHGAP25 NM_014882	F: 5′-CTGAGAGACGCTTTTGATGCT-3′ R: 5′-TCTCGGAGGTAGAGCTTTAACA-3′
GABRD NM_000815	F: 5′-GCATCCGAATCACCTCCACTG-3′ R: 5′-GATGAGTAACCGTAGCTCTCCA-3′
GAPDH	F: 5′-GGAGCGAGATCCCTCCAAAAT-3′
NM_001256799	R: 5′-GGCTGTTGTCATACTTCTCATGG-3′

### Western blotting

Total protein from OS cell lines and hFOB1.19 was isolated using RIPA buffer (Beyotime, China). Protein samples (20 μg each) were then separated using SDS-PAGE and transferred onto PVDF membranes. Following this, the membranes were blocked using 5% non-fat milk in TBST for 1 hour at room temperature and subsequently incubated with specific primary antibodies at 4˚C overnight. The employed antibodies included: CD37 (Abcam, ab300400, 1:1,000), ARHGAP25 (Abcam, ab181202, 1:10,000), GABRD (Abcam, ab93619, 1:1,000), β-Tubulin (Cell Signaling Technology, #2146, 1:1,000), and GAPDH (Cell Signaling Technology, #5174, 1:1,000).

### Statistical analysis

The statistical software R version 4.1.2 and Prism 8.0 (GraphPad) were utilized to conduct data analysis and generate visual representations of the findings. To assess the associations between variables, Spearman or Pearson correlation coefficients were calculated. Kaplan-Meier methodology was utilized to construct survival curves, and the log-rank test was performed to compare the curves. Univariable and multivariable Cox regression models were used to identify prognostic factors for overall survival. Experimental data were presented as mean ± standard error of the mean (SEM). One-way ANOVA followed by Tukey *post hoc* analysis was used to compare 3 or more groups. *P* values of <0.05 were considered to be statistically significant.

## Results

### Construction of M1 macrophage-related gene signature

**Figure 1:**
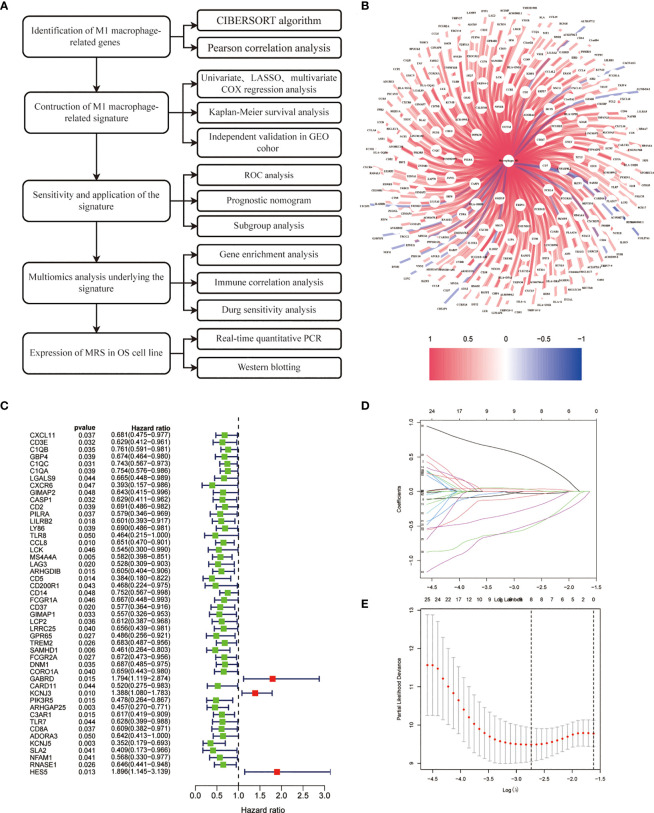
Construction of risk prognostic model. **(A)** Flowchart of the present research. **(B)** M1 macrophage-related genes. **(C)** Univariate Cox regression analysis obtained 49 candidate prognostic MRGs for OS. **(D)** LASSO regression analysis. **(E)** Selection of the optimal penalty parameter for LASSO regression.

**Figure 1** illustrates the study’s workflow. The CIBERSORT algorithm was employed to analyze the M1 macrophage subpopulation abundance in each sample. A total of 281 genes exhibiting a correlation with M1 macrophages were pinpointed through Pearson correlation analysis (|R2| > 0.4, p < 0.001) (**Figure 1**, **Supplementary Table S1**). Forty-nine genes associated with M1 macrophages were recognized as potential prognostic indicators via univariate Cox analysis (**Figure 1**). To mitigate the risk of overfitting, LASSO Cox regression was subsequently performed (**Figures 1A–E**). After applying the LASSO algorithm, the multivariate Cox regression model was used to identify the final gene set, which consisted of 3 robust genes, forming a prognostic signature for overall survival.

### Correlation between MRS and prognosis of OS patients

**Figure 2:**
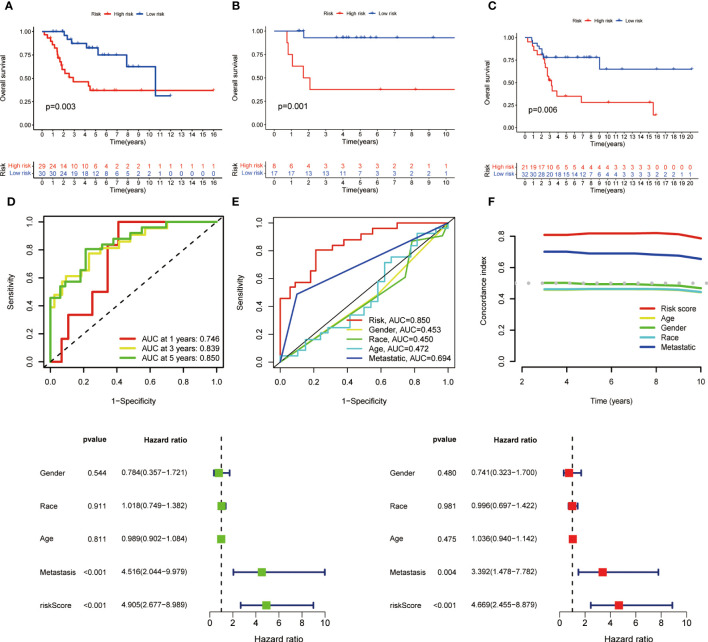
Kaplan-Meier survival analysis in training and validation sets and prognostic value of MRS. **(A)** Kaplan-Meier analysis of the overall survival of training set. **(B)** Kaplan-Meier analysis of the overall survival of internal validation set. **(C)** Kaplan-Meier analysis of the overall survival of external validation set. **(D)** ROC curve and AUCs at 1-year, 3-years and 5-years survival for MRS. **(E)** The ROC curve of the risk score and clinicopathological variables. **(F)** C index of the risk score and clinicopathological variables. **(G)** Forest plot for univariate Cox regression analysis. **(H)** Forest plot for multivariate Cox regression analysis.

Coefficients of the three M1 macrophage-associated genes were employed to determine scores for each patient. The risk score computation was as follows: Risk score = (-2.284 × CD37 expression) + (3.845 × GABRD expression) + (-3.632 × ARHGAP25 expression). Subsequently, participants were assigned to low- or high-risk groups based on the median value of the risk score. In the training set, high-risk patients demonstrated shorter overall survival than low-risk counterparts (p < 0.001, **Figure 2**). Similar trends were observed in both internal and external validation cohorts (p < 0.001, **Figures 2A–C**).

To assess the signature’s effectiveness, a time-dependent ROC curve was utilized. AUC values for 1-year, 3-year, and 5-year survival periods were 0.746, 0.839, and 0.850, respectively (**Figure 2**). The one-year survival rate AUC suggested that both risk score (0.850) and metastasis (0.694) had satisfactory predictive capabilities (**Figure 2**). As depicted in **Figure 2**, the risk score’s C-index surpassed that of clinicopathological factors.

Univariate and multivariate Cox regression analyses were performed to evaluate the prognostic value of risk score and other factors. The risk score and metastasis emerged as significant independent prognostic factors, as evidenced by the HR and CI values: 4.905 (95% CI = 2.677–8.989, p < 0.001) and 4.516 (95% CI = 2.044-9.979, p < 0.001) for univariate analysis, and 4.669 (95% CI = 2.455-8.879, p < 0.001) and 3.392 (95% CI = 1.478-7.782, p = 0.004) for multivariate analysis (**Figures 2A–H**).

### Independent prognostic value of the MRS and establishment of the nomogram

**Figure 3:**
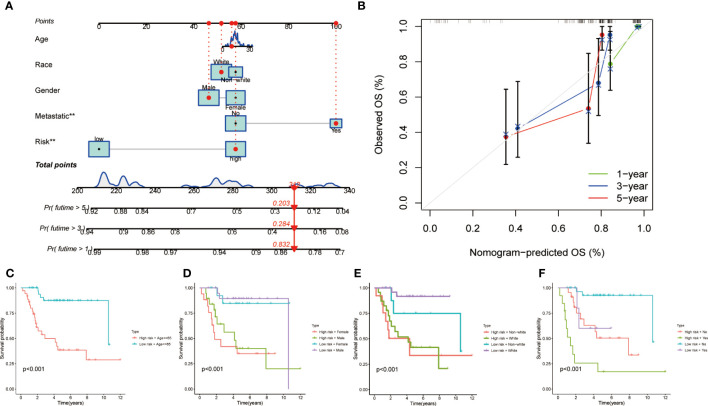
Clinical prognostic nomogram for survival prediction and subgroup analysis. **(A)** A nomogram combining clinicopathological variables and risk score predicts 1, 3, and 5 years OS of HCC patients. **(B)** Calibration plots for 1-, 3-, and 5-years survival predictions. **(C-F)** Subgroup survival analysis in the high- and low-risk groups, **(C)** Age ≤65y **(D)** Gender between male and female patients **(E)** Race between white and non-white **(F)** Metastasis or not.

In order to furnish a numerical means of clinical utilization, a nomogram was devised incorporating factors such as age, gender, race, metastasis, and risk score to forecast the overall survival of patients (**Figure 3**). The calibration plot demonstrates a high degree of agreement between the observed and predicted rates of 1, 3, and 5-year overall survival (**Figure 3**). Finally, the applicability of MRS was evaluated by grouping patients based on their age, gender, race and metastasis. The results showed that patients with high-risk scores within each subgroup had an unfavorable prognosis, demonstrating the efficacy of MRS in predicting outcomes for all patients (**Figures 3A–F**).

### Functional analysis of high- and low-risk score groups

**Figure 4:**
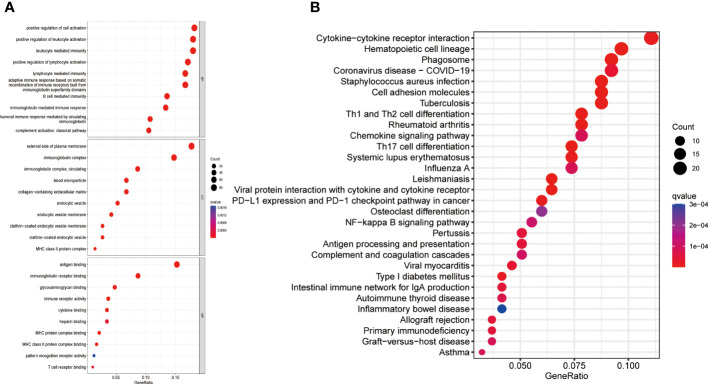
Gene enrichment in high- and low-risk groups. **(A)** GO enrichment analysis **(B)** KEGG enrichment analysis. GO, Gene Ontology; KEGG, Kyoto Encyclopedia of Genes and Genomes; BP, Biological process; CC, Cellular component; MF, Molecular function.

A comprehensive analysis of GO and KEGG methodologies was carried out to explore the potential mechanisms responsible for the differing prognoses observed between high- and low-risk groups. A total of 631 differentially expressed genes were identified between the two groups. GO enrichment analysis of biological processes (BP) indicated that the DEGs were predominantly involved in “leukocyte activation regulation,” “positive modulation of lymphocyte activation,” and “leukocyte-driven immunity.” In relation to cellular components (CC), the DEGs were chiefly linked to “immunoglobulin complexes,” “external aspect of plasma membrane,” and “circulating immunoglobulin complexes.” Likewise, molecular function (MF) analysis revealed that the DEGs primarily focused on “antigen binding” and “immunoglobulin receptor binding” (**Figure 4**). KEGG analysis results showed that the DEGs were mainly enriched in several pathways, including “Th1 and Th2 cell differentiation”, “Cytokine-cytokine receptor interactions,” “Antigen processing and presentation,” “Osteoclast differentiation,” “PD-L1 expression and PD-1 checkpoint pathway in cancer”, and “NF-kappa B signaling pathway” (**Figure 4**).

### Immune-related analysis of high- and low-risk score groups

**Figure 5:**
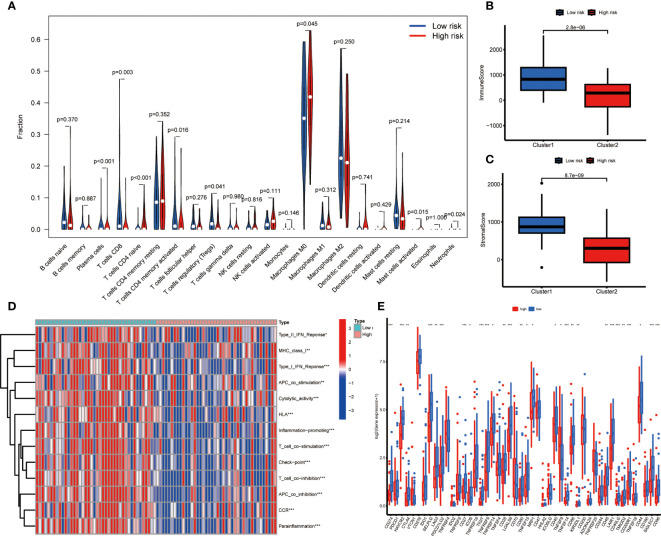
Immune related analysis in high- and low-risk groups. **(A)** Differences in the infiltration of immune cells between the high- and low- risk groups. **(B, C)** Comparison of immune score **(B)**, and stromal score **(C)** between the high- and low-risk groups. **(D)** The correlation between the signature and immune functions. **(E)** Differential expression of immune checkpoint genes between the high- and low-risk groups *P < 0.05; **P < 0.01; ***P < 0.001.

In order to further investigate the relationship between risk score and infiltration of immune cells into tumors, the CIBERSORT algorithm was employed to compare the proportions of 22 different types of immune cells between groups of individuals classified as either low or high risk. The findings revealed that the low-risk group had higher fractions of plasma cells, CD8+ T cells, regulatory T cells, and memory CD4+ T cells (p < 0.05), while the high-risk group had higher fractions of M0 macrophages, which is associated with immunosuppressive activity (p < 0.05) (**Figure 5**). There was an indication of a greater prevalence of immune and stromal cells within the low-risk group according to the ESTIMATE algorithm (**Figures 5A–C**). The ssGSEA algorithm was utilized to deduce the immune function. **Figure 5** reveals that there exists a significant disparity between the two groups in immune function. These findings suggest that the low-risk group exhibits a higher level of immune function activity. Then we investigated the potential association between risk scores and the expression levels of immune checkpoint genes. Patients categorized as low-risk exhibited significantly elevated expression levels of 26 immune checkpoint genes, namely CD274, HAVCR2, SELPLG, LAG3, CD27, ICOS, TIGHT, TNFRSF9, TNFRSF14, CD28, LGALS9, CD80, TNFRSF15, NRP1, CD40, TNFSF14, CD86, KIR3DL1, CD48, LAIR1, CD40LG, TMIGD2, CD200R1, CD44, CD96, SIGLEC7, while TNFSF9 was found elevated expression in the high-risk group (**Figure 5**).

### Drug response features of MRS in OS

We investigated the correlation between the risk score and the effectiveness of targeted therapy and chemotherapy for OS patients. The findings indicated a positive correlation between the IC50 of Dasatinib and the risk score. In contrast, a negative correlation was observed between the IC50 of Daporinad, Linsitinib, Sabutoclax, and Dihydrorotenone and the risk score (**Supplementary Figure S1**).

### Expression of MRGs in different OS cell lines

**Figure 6:**
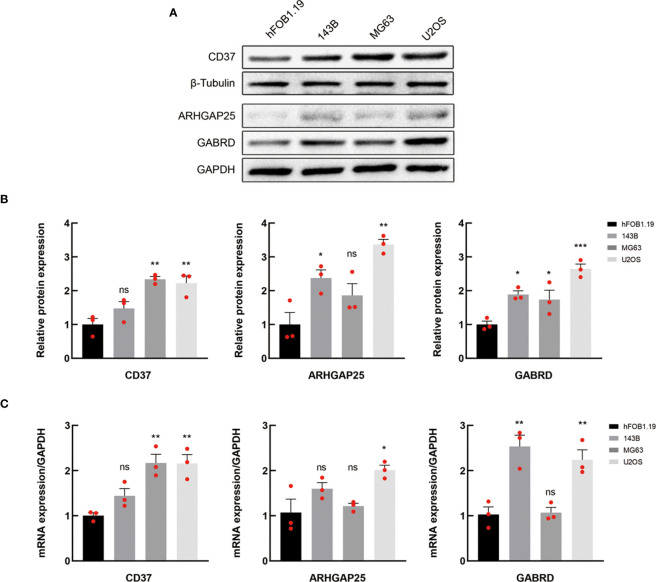
Verification of the expression of MRGs. **(A, B)** Western blotting of CD37, ARHGAP25 and GABRD proteins in normal and OS cell lines. **(C)** The mRNA expression levels of the above genes in normal and OS cell lines were analyzed by qRT-PCR. In **(A)** data are representative of three independent experiments. In B and C, data are presented as mean ± SEM of three independent experiments; ns, not significant; *P < 0.05; **P < 0.01; ***P < 0.001 compared with hFOB1.19 normal cells, as analyzed by ANOVA.

In order to investigate the disparities in gene expression patterns between tumor and normal cell lines, we chose three OS cell lines to determine their expression levels of mRNA and protein, with the normal osteoblast hFOB1.19 serving as the control group. Western blotting results indicate that CD37 protein expression was markedly higher in MG63 and U2OS cell line when compared with normal osteoblast hFOB1.19, and GABRD expression levels were significantly upregulated in U2OS cell line. In addition, the protein expression levels of ARHGAP25 were elevated in both 143B and U2OS cell lines. **Figures 6A** illustrate these findings.

The mRNA expression levels of CD37, GABRD and ARHGAP25 were analyzed by qRT-PCR (**Figure 6**). The outcomes revealed that the levels of CD37, GABRD and ARHGAP25 were significantly elevated in the tumor cells than that in the normal cell line.

## Discussion

Osteosarcoma is an infrequent malignant neoplasm of bone tissue that primarily afflicts the adolescent and young adult demographic. The prevailing treatment modality involves neoadjuvant chemotherapy, surgery, and adjuvant chemotherapy (28). This study generated a signature for M1 macrophages and subsequently evaluated for its correlation with overall survival in patients with osteosarcoma. Additionally, the potential impact of this signature on the tumor microenvironment and its response to therapy were investigated. To explore the underlying mechanisms involved, gene enrichment analysis was conducted.

The gene ontology functions analysis showed that the M1 macrophage-related genes pertained largely to the immune system, encompassing positive regulation lymphocyte activation, immunoglobulin complex and antigen binding. Among the biological processes, cell activation, including lymphocyte stood out as the most significantly correlated term. Lymphocyte activation is a crucial mechanism that triggers the lymphocytes to recognize and attack cancer cells. The level of lymphocyte activation has been found to be associated with the prognosis of patients with cancer. A study by Galon et al. found that patients with colorectal cancer who had a high density of activated T lymphocytes had a better prognosis than those with a low density of activated T lymphocytes (29). Similarly, another study showed that patients with ovarian cancer who had a high level of tumor-infiltrating lymphocytes had a better prognosis than those with a low level of tumor-infiltrating lymphocytes (30). This suggests that the immune response mediated by lymphocytes plays a critical role in determining the outcome of cancer patients. Furthermore, the level of lymphocyte activation has also been found to be a predictor of response to immunotherapy. For instance, patients with melanoma with a higher T-cell activation level had a better response to immune checkpoint inhibitors than those with lower levels of T-cell activation (31). Through KEGG analysis we found that many of them were related to cytokine-cytokine receptor interaction, the nuclear factor kappa B (NF-κB) signaling pathway and osteoclast differentiation. Cytokines are recognized as essential regulators of both innate and adaptive immune systems, facilitating communication among immune cells via paracrine and autocrine signaling mechanisms (32). Interactions between cytokines and their receptors are acknowledged as crucial determinants of inflammation and oncogenesis (33). These results suggested that disparities in immune function and carcinogenesis and progression exist between the high- and low-risk cohorts, subsequently influencing the prognosis of patients within the two groups.

The results of tumor microenvironment in the two groups revealed that patients with low-risk scores exhibited a higher proportion of CD8+ T cells, regulatory T cells, and memory CD4+ T cells and lower proportion of M0 macrophage. This observation indicates the ability of MRS to differentiate the tumor microenvironment. As we know, CD8+ T cells play an important role in the immune response against cancer and serve as the fundamental component of contemporary efficacious cancer immunotherapies (34). The CIBERSORT algorithm was employed to analyze the comparative distribution of infiltrating immune cell subtypes across various tumor specimens. Our findings evinced a positive association between both immune score and estimate score with the low-risk cohort. The ssGSEA algorithm showed that patients in the low-risk group exhibited a significantly elevated cytolytic activity score. This finding might imply a potentially favorable tumor immune microenvironment. Also, the results showed a higher expression level of immune checkpoint genes. OS is recognized as a highly heterogeneous cancer type, and to date, the efficacy of immunotherapy for OS remains unsatisfactory (35). The MRS might help to identify patients suitable for immunotherapy.

Three genes were used to establish the prognostic model. CD37 is a transmembrane protein that plays a crucial role in the regulation of tumorigenesis and progression (36). Moreover, CD37 serves as a significant immune marker for various immune cells, such as T-cells, B-cells, and macrophages. Its high expression may suggest the adequate filtration of immune cells and immunity in the tumor microenvironment (37). GABRD is a gene that codes for a protein called gamma-aminobutyric acid (GABA) receptor delta subunit (38). Study has investigated the relationship between GABRD and cancer. Niu et al. found that GABRD was overexpressed in colorectal cancer tissues and that higher levels of GABRD were associated with poorer prognosis in colorectal cancer patients (39). ARHGAP25 has been reported in study of OS. Ding et al. found that ARHGAP25 exerted an inhibitory effect on MG63 cell proliferation, migration, and progression of epithelial–mesenchymal transition (EMT) and could work as a predictive biomarker for osteosarcoma metastasis (40). These genes play an important role in TME and tumorigenesis and progression, which could predict prognosis of OS.

The present study had some limitations. First, we constructed and validated the prognostic model with a single retrospective data source. Second, the sample size was not large enough. Third the database provides limited clinical information. Thus, a prospective study is needed to verify the predictive value of the signature.

In summary, utilizing a macrophage genes signature demonstrates efficacy in predicting both the prognosis and therapy response of OS. Additionally, immune analysis confirms a correlation between the risk score and the tumor microenvironment. Our findings, therefore, provide a cogent account for the disparate prognoses observed among patients and furnish a justification for further inquiry into biomarkers and anti-tumor treatment strategies.

## Data availability statement

Publicly available datasets were analyzed in this study. This data can be found here: The Cancer Genome Atlas (https://portal.gdc.cancer.gov/) and the Gene Expression Omnibus (GEO) database (https://www.ncbi.nlm.nih.gov/geo/) for the GSE21257 dataset.

## Ethics statement

TCGA and GEO belong to public databases. The patients involved in the database have obtained ethical approval. Users can download relevant data for free for research and publish relevant articles. Our study is based on open source data, so there are no ethical issues and other conflicts of interest.

## Author contributions

XM and FS: design and conduct experiments, data analysis, prepared and revised the manuscript. JJ, BZ and PD: carry out some experiments and data analysis. MS and WX: statistical analysis. LW and YK: overall supervision, design, funding support and manuscript preparation and substantively revision. All authors read and approved the final manuscript.
