# Supplementary figures and images for: Prognostic and immunological significance of an M1 macrophage-related gene signature in osteosarcoma
Source: Front Immunol. 2023 Jul 3;14:1202725. doi: 10.3389/fimmu.2023.1202725 (PMC10350629; doi:10.3389/fimmu.2023.1202725)

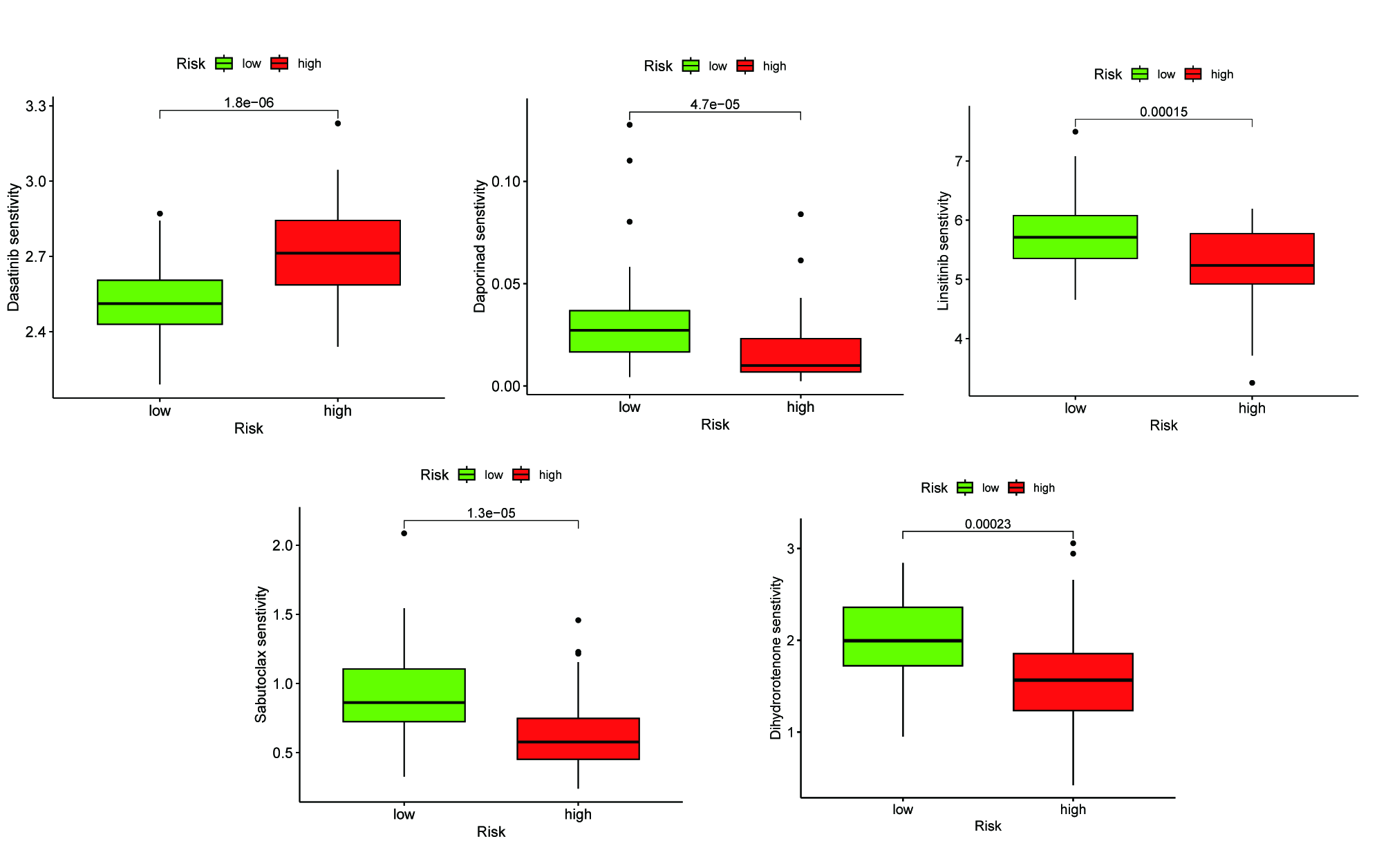

Supplement: Supplementary file 1 [file Image_1.tif]
